# Cadaveric Report of an Unusual Axillary Artery Branching Variation: A Potential Compression Site for the Lateral Root of the Median Nerve

**DOI:** 10.7759/cureus.86064

**Published:** 2025-06-15

**Authors:** Kamal A Abouzaid, Hannah J Grimmett, Ketsia Kimbimbi, Niharika Dar, Ava Greenberg, Hadiseh Faridi Tavana, Ahmad Imam

**Affiliations:** 1 Department of Anatomical Sciences, William Carey University College of Osteopathic Medicine, Hattiesburg, USA

**Keywords:** brachial plexus, case report, entrapment, median nerve, vascular nerve compression

## Abstract

The median nerve is formed by the union of lateral and medial roots originating from the lateral and medial cords of the brachial plexus, respectively. The lateral root of the median nerve is responsible for motor innervation to some of the anterior forearm muscles as well as sensory innervation to the lateral palm and the palmar surfaces of the lateral three-and-a-half digits. While anatomical variations in the axillary artery’s branching pattern are relatively common, vascular compression of the median nerve, particularly its individual roots, is exceedingly uncommon. In this report, we observed that the distal part of the axillary artery gave rise to a single large-caliber common trunk. The lateral root of the median nerve was found to be clamped between the distal axillary artery and its common trunk branch, which formed a dynamic acute arterial angle that narrowed further upon abduction of the arm. At 90 degrees of abduction, the angle was obliterated; this resulted in direct compression of the nerve root between the two arteries. No atrophy was noted in the muscles of the forearm, suggesting the compression may have been intermittent or subclinical. Additionally, the tortuosity of the proximal brachial artery observed in this case may reflect long-term compensatory changes due to restricted arterial mobility. This case highlights a previously unreported potential site of compression of the lateral root of the median nerve by a vascular anomaly. The findings underscore the importance of recognizing such variations during clinical assessments and surgical procedures involving the axilla.

## Introduction

The median nerve is formed by the union of a lateral root and a medial root, which arise from the lateral and medial cords of the brachial plexus, respectively. It contains fibers from all five spinal nerve roots (C5-T1) with the lateral root carrying fibers from C5-C7 and the medial root carrying fibers from C8-T1. Functionally, the median nerve innervates for the majority of the muscles in the anterior compartment of the forearm including pronator teres (C6-C7), flexor carpi radialis (C6-C7), flexor digitorum superficialis (C8-T1), palmaris longus (C7-C8), lateral half of flexor digitorum profundus (C8-T1), flexor pollicus longus (C7-C8), and pronator quadratus (C7-C8). In the hand, it provides innervation to flexor pollicis brevis (C8-T1), abductor pollicis brevis (C8-T1), opponens pollicis (C8-T1), and the lateral two lumbricals (C8-T1). With respect to its sensory functions, the median nerve supplies the lateral aspect of the palm (C6-C7), the palmar aspects of the thumb (C6), and the index, middle, and lateral half of the ring fingers (C7). Anatomically, the lateral and medial roots of the median nerve typically unite anterior to the distal part of the axillary artery; it then descends lateral to the brachial artery and shifts medial to the artery at the level of insertion of the coracobrachialis [1].

Within the axillary sheath, the axillary artery and vein are closely related to the brachial plexus and its branches. The axillary artery gives rise to multiple branches along its course: the superior thoracic artery arises proximal to pectoralis minor; the thoracoacromial and lateral thoracic arteries originate deep to pectoralis minor; and the subscapular and anterior and posterior circumflex humeral arteries arise distal to pectoralis minor [1]. Anatomical variation in the branching pattern of the axillary artery is a relatively common finding, and many different In the present case, we report an unusual anatomical variation in which the lateral root of the median nerve was clamped between the distal axillary artery and a common trunk branch arising from the third part of the axillary artery.variations have been observed and described [2,3]. For instance, a common trunk giving rise to both the subscapular and the posterior circumflex humeral arteries occurs in up to 30% of cases [4]. Further, there is a significant amount of variation that can occur in the third part of the axillary artery; most commonly, the two circumflex humeral arteries may arise from a common trunk [3].

Compression of the median nerve along its course is a well-documented clinical issue. It can be compressed in the carpal tunnel or by nearby structures such as a muscle, ligament, or blood vessel. Carpal tunnel syndrome, the most common form of median nerve compression, is seen in approximately 1.55% of the US population, the equivalent of 2.65 million people [5-6]. Aside from entrapment at the wrist, the median nerve can be compressed along various points of its course by other structures such as the brachialis muscle, Struthers’ ligament, the bicipital aponeurosis, pronator teres, flexor digitorum superficialis, vascular structures in the forearm, and the accessory head of flexor pollicis longus [7]. While muscle compression of the median nerve is relatively common, vascular abnormalities resulting in median nerve compression are rarely reported [8]. 

In the present case, we report an unusual anatomical variation in which the lateral root of the median nerve was clamped between the distal axillary artery and a common trunk branch arising from the third part of the axillary artery. This unique relationship may result in neurologic symptoms and highlights the clinical significance of recognizing vascular variations in relation to neural structures. 

## Case presentation

This report describes an unusual vascular variation in the branching pattern of the axillary artery, which may have had a compression effect on the lateral root of the median nerve. The anomaly was identified during a pedagogical dissection of the axilla and its contents as part of a first-year clinical anatomy course at William Carey University College of Osteopathic Medicine. The cadaveric donor was an 85-year-old male Caucasian who was obtained through the University of South Alabama Anatomical Gift Program. The cause of death, as noted on the death certificate, was myelocytic leukemia. 

**Figure 1:**
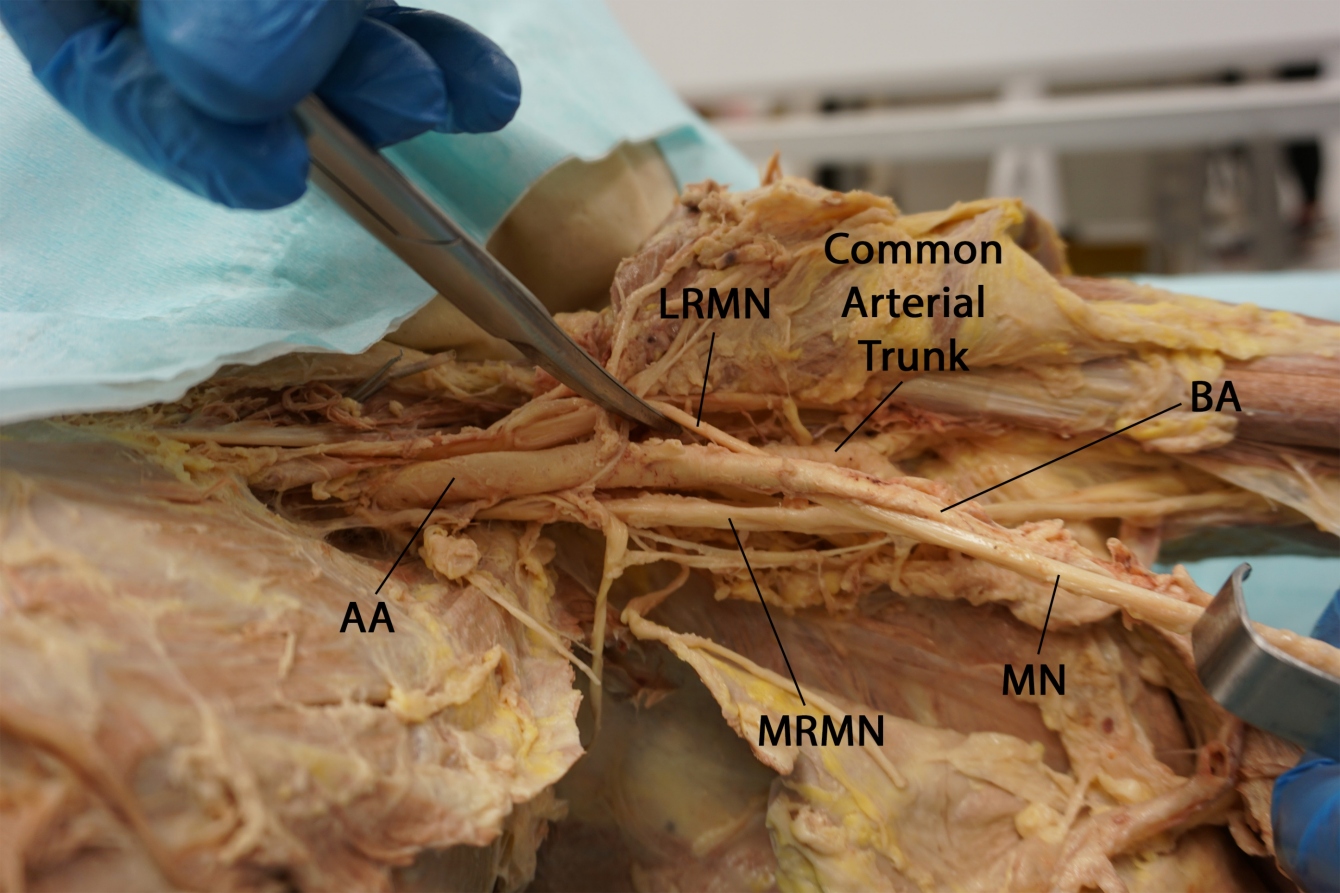
Inferior view of the left axilla showing the lateral root of the median nerve passing between the axillary artery and its common trunk and joining the medial root of the median nerve posterior to the distal part of the axillary artery. (AA: axillary artery, LRMN: lateral root of median nerve, MRMN: medial root of median nerve, MN: median nerve, BA: brachial artery)

**Figure 2:**
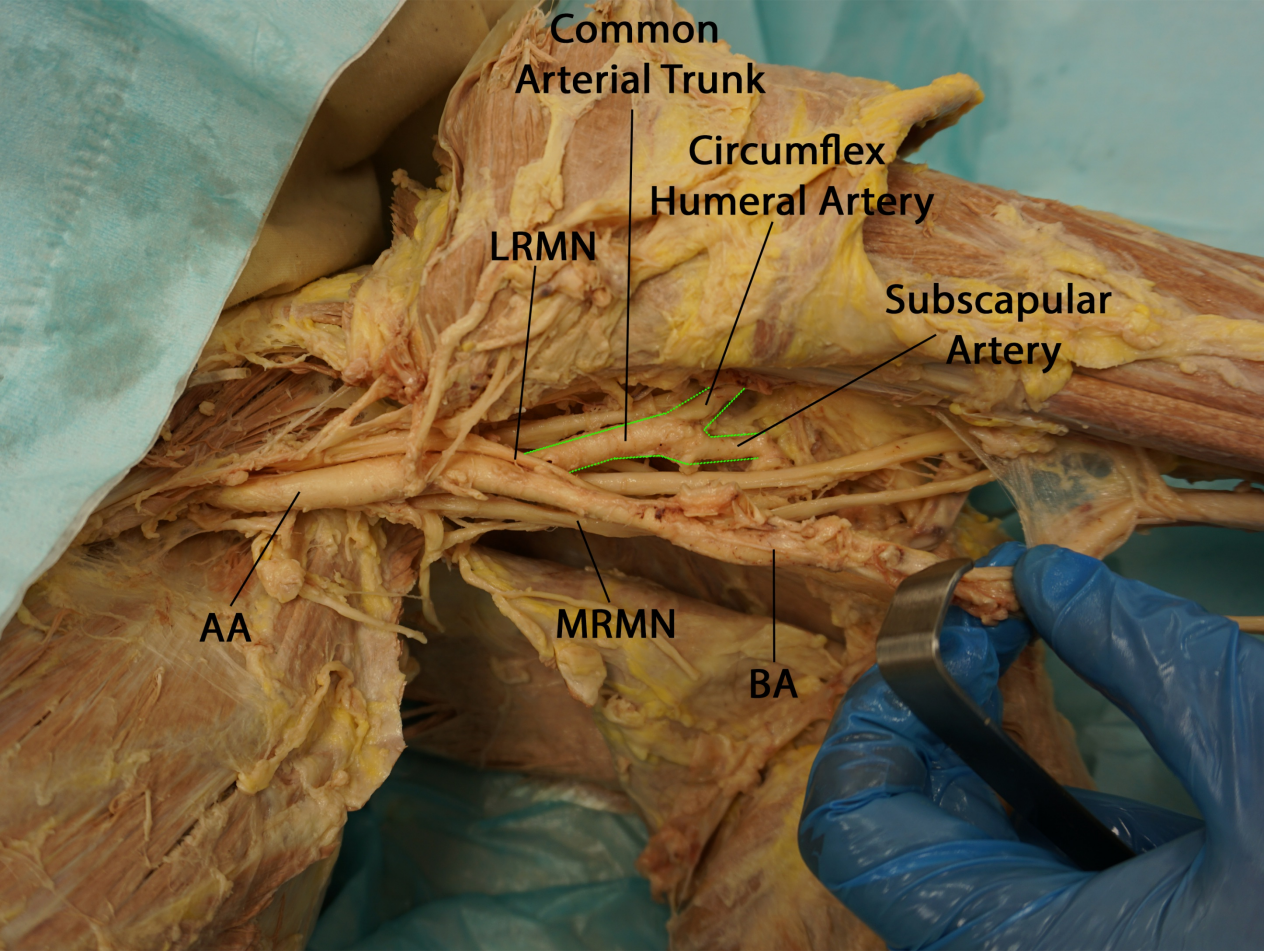
Anteroinferior view of the left axilla demonstrating the thickness of the common arterial trunk and its bifurcation into the circumflex humeral artery and the subscapular artery. The tortuous initial segment of the brachial artery has been retracted to expose the angle between the axillary artery and its common trunk branch. (AA: axillary artery, LRMN: lateral root of median nerve, MRMN: medial root of median nerve, BA: brachial artery)

During the dissection of the infraclavicular portion of the brachial plexus and the axillary artery, we observed a notable variation in the vascular anatomy of the left axilla. Specifically, the typical branches of the third part of the axillary artery arose as a common arterial trunk that passed beneath the lateral root of the median nerve (Figures 1, 2). 

**Figure 3:**
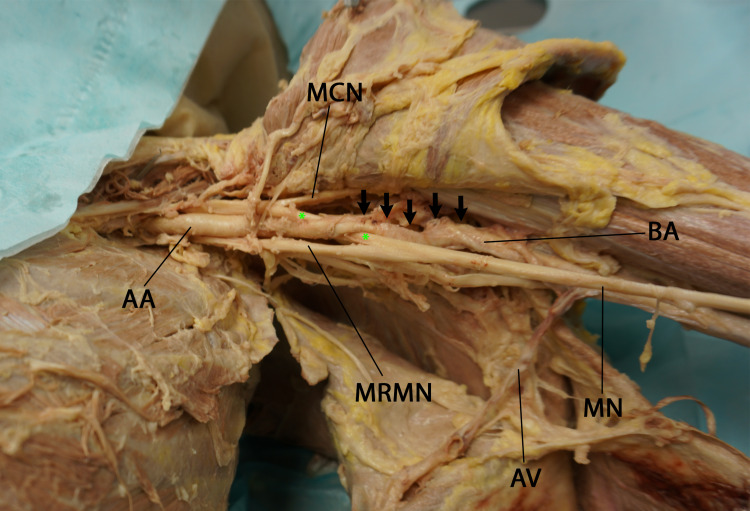
Anteroinferior view of the left axilla showing the tortuous initial segment of the brachial artery (arrows) with pectoralis minor retracted laterally. (AA: axillary artery, MCN: musculocutaneous nerve, MRMN: medial root of median nerve, MN: median nerve, BA: brachial artery, asterisks: lateral root of median nerve, AV: axillary vein)

The common arterial trunk, which was notably larger in caliber than the brachial artery, gave rise to a single circumflex humeral artery, which then coursed through the quadrangular space in a manner similar to the typical path of the posterior circumflex humeral artery. The common arterial trunk then continued as the subscapular artery. After giving off the common arterial trunk branch, the axillary artery continued as the brachial artery. The proximal portion of the brachial artery exhibited a tortuous course for approximately two inches (Figure 3). 

**Figure 4:**
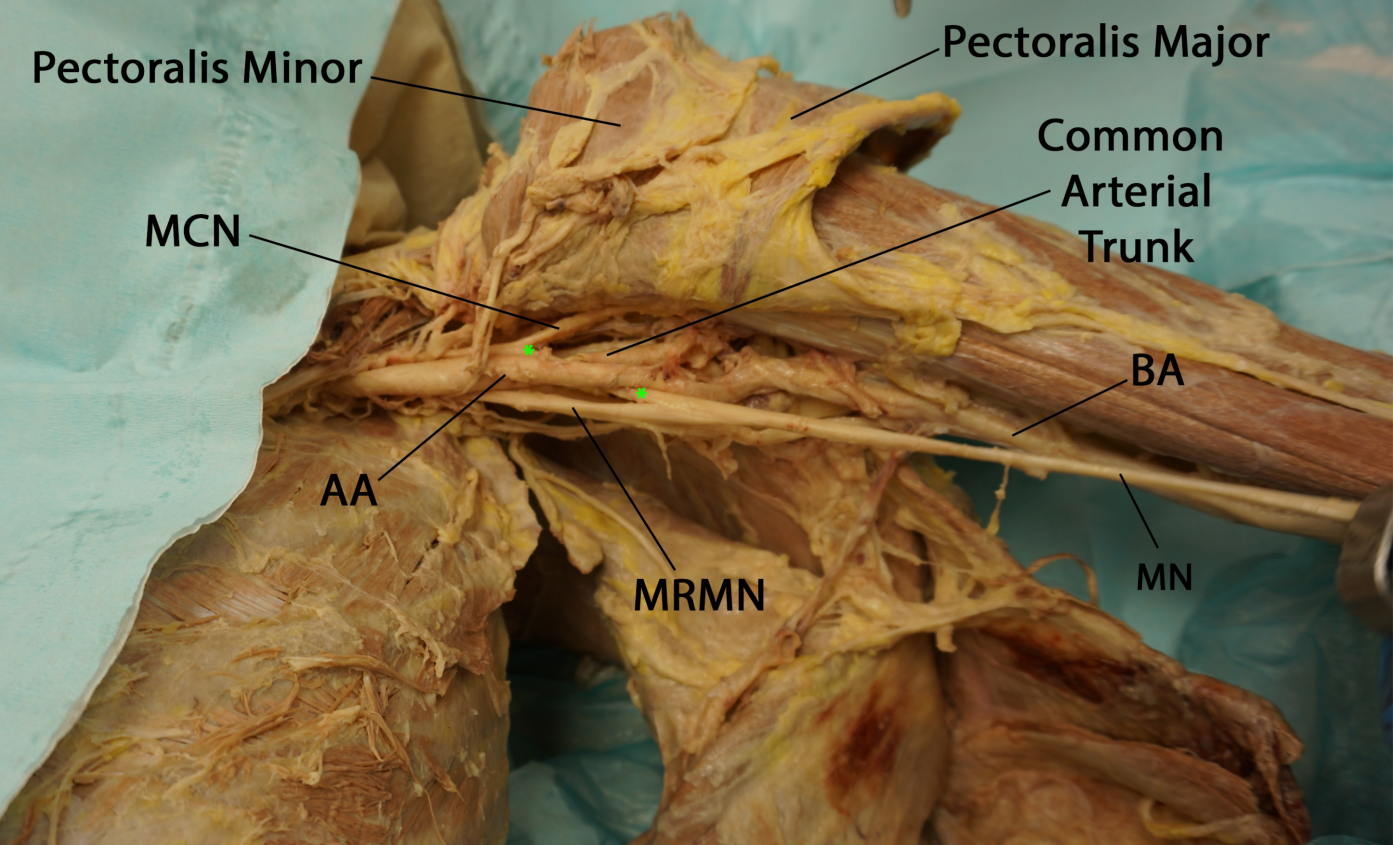
Anteroinferior view of the left axilla illustrating compression of the lateral root of the median nerve during arm abduction. (MCN: musculocutaneous nerve, AA: axillary artery, MRMN: medial root of median nerve, BA: brachial artery, MN: median nerve, asterisks: lateral root of median nerve)

Further careful dissection of the brachial plexus revealed that the lateral cord gave rise to the lateral root of the median nerve and continued as the musculocutaneous nerve. The lateral root of the median nerve traversed medially over the common arterial trunk and passed posterior to the axillary artery through the acute angle formed between the third part of the axillary artery and its single branch, the common arterial trunk. This angle was dynamic; it decreased as the arm was abducted, and, at 90 degrees of abduction, the angle was obliterated. In this position, the axillary artery and its common arterial trunk branch were brought into close contact, resulting in the compression of the lateral root of the median nerve between them (Figure 4).

**Figure 5:**
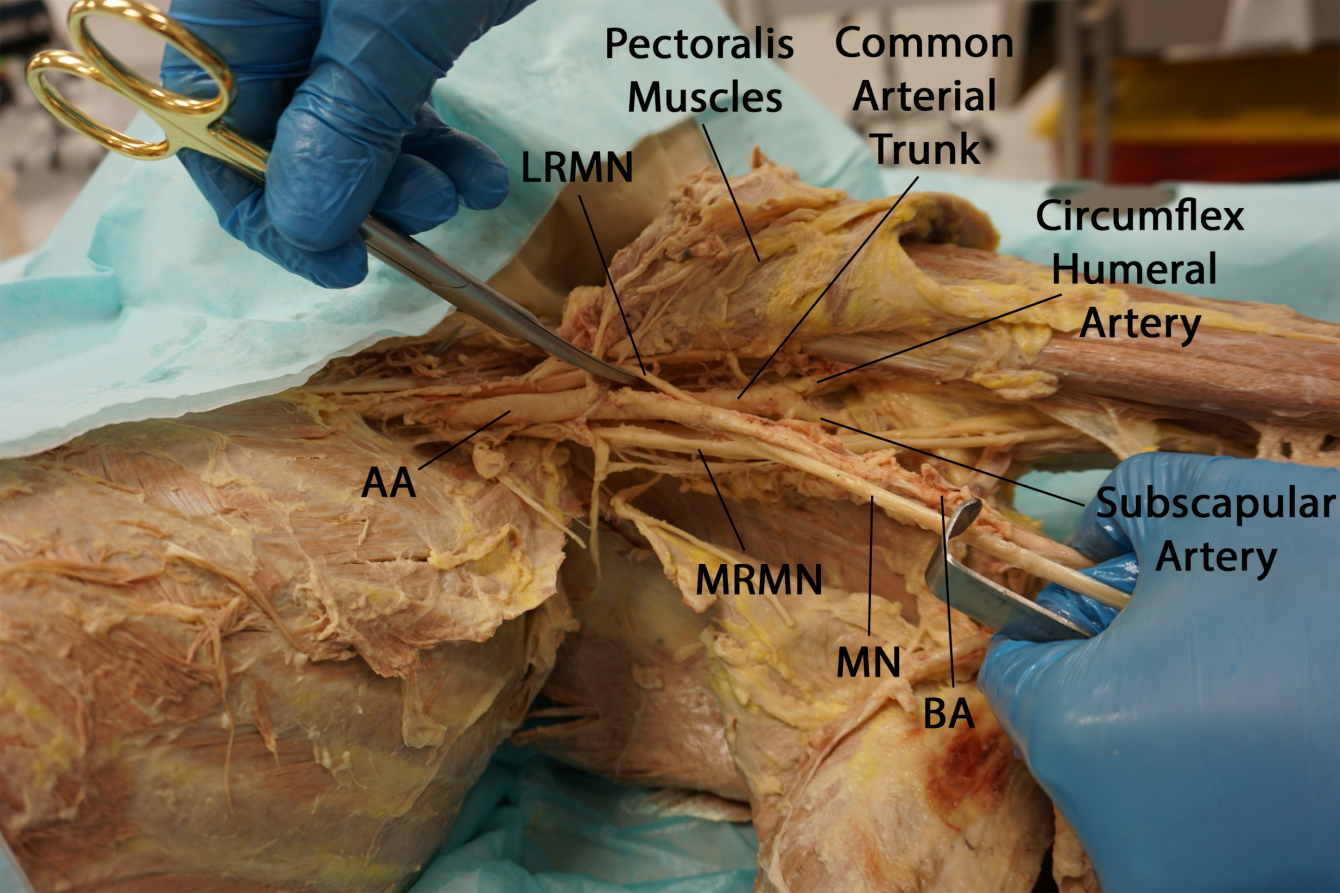
Inferior view of the left axilla showing the lateral and medial roots of the median nerve joining posterior to the axillary artery and continuing along the medial aspect of the brachial artery as the median nerve. (AA: axillary artery, LRMN: lateral root of median nerve, MRMN: medial root of median nerve, MN: median nerve, BA: brachial artery)

Deep into the axillary artery, the lateral and medial roots of the median nerve converged to form the median nerve. The nerve then descended along the medial aspect of the brachial artery (Figure 5). 

Notably, no other anatomical anomalies were detected in this case. Additionally, an examination of the muscles in the anterior compartment of the left forearm revealed normal muscle sizes, in comparison to the right forearm, which suggests no signs of nerve compression-related atrophy.

This case highlights the clinical significance of vascular anomalies and their potential impact on adjacent nerves and underscores the importance of detailed anatomical knowledge in understanding the possible complex variations that may contribute to clinical presentations.

## Discussion

Vascular anomalies of the axillary artery are well documented in the literature. George et al. [2] described a case in which the axillary artery bifurcated into almost two equal-caliber trunks where one superficial branch continued as the brachial artery and one deep branch that divided into a profunda brachii artery and a common trunk that further bifurcated into a common circumflex humeral artery and subscapular artery. Other abnormal branching patterns of the axillary artery have also been documented. Magden et al. [9], for example, investigated a case in which the serratus anterior muscle was supplied by a direct branch of the first part of the axillary artery.

Although such vascular anomalies are relatively common, compression of the median nerve by vascular structures is rare [8]. Lobo et al. [10] presented a case of median nerve compression due to a brachial artery pseudoaneurysm, in which the patient exhibited symptoms involving the left median nerve territory. In this case report, we observed the lateral root of the median nerve being clamped between the distal axillary artery and its common trunk branch. This anatomical configuration created an angle between the axillary artery and its common branch that became obliterated when the arm was abducted to 90 degrees, leading to compression of the lateral root of the median nerve. As a result, it is reasonable to expect symptoms associated with median nerve compression would be exacerbated during arm abduction or overhead arm movement.

Although no previous literature describes vascular compression of the lateral root of the median nerve specifically, similar mechanisms have been reported. Kim et al. [11] reported a case of axillary arteriovenous malformation compressing the median nerve. The patient presented with a slow-growing mass in the right axilla and neurogenic pain, which worsened with abduction and external rotation of the arm and radiated from their forearm to their fingers. In that case, the median nerve passed between the arteriovenous malformation involving both the axillary artery and vein. While the mechanism was vascular in nature, it involved the entire median nerve rather than one of its roots as seen in our case. Nonetheless, the clinical presentation described by Kim et al. supports our hypothesis that abduction in the presence of such vascular anomaly can lead to neurogenic pain.

Our case is unique in that only the lateral root of the median nerve could potentially be compressed between the two arteries. Given the fibers it carries, this could manifest in a complex presentation of neurogenic symptoms affecting C5, C6, and C7 distribution of the median nerve. This compression may potentially affect the function of some of the muscles of the anterior compartment of the forearm and may cause sensory impairment of the lateral palm and the palmar surfaces of the lateral three digits. The symptoms could manifest as muscle pain, weakness, and sensory disturbances such as numbness or tingling, particularly worsened by arm abduction.

Vascular compression of adjacent nerves is a well-known phenomenon. For example, trigeminal nerve compression commonly due to the superior cerebellar artery (SCA) is thought to cause trigeminal neuralgia [12]. This condition presents as a facial-pain syndrome characterized by unilateral, shock-like pain following the sensory distribution of the trigeminal nerve [13]. In a prospective study of patients with trigeminal neuralgia, Chen et al. [14] reported arterial compression was observed in 68.7% of the cases. Similarly, glossopharyngeal neuralgia, characterized by acute stabbing pain triggered by swallowing, is also speculated to result from compression by nearby arteries [15]. In a study of 10 patients with glossopharyngeal neuralgia, Hiwatashi et al. [16] found that each patient had either the posterior inferior cerebellar artery (PICA) or anterior inferior cerebellar artery (AICA) in contact with the glossopharyngeal nerve root. These examples underscore the potential for arteries to cause localized nerve compression and neurogenic pain. In the context of our case, the unusual anatomical position of the lateral root of the median nerve between the axillary artery and its common trunk branch may similarly result in neurogenic symptoms. 

Another noteworthy finding in this case was the tortuosity in the proximal segment of the brachial artery. Tortuous arteries are generally seen in areas requiring uninterrupted blood supply during movement and growth [17]. One of the more notable tortuous vessels in the body is the facial artery. Its tortuosity accommodates the dynamic stretching that occurs during mastication [18]. The tortuosity of the facial artery is directly proportional to mandibular use. Indeed, its tortuosity increases with age, as demonstrated by Soikkonen et al. [19].

While peripheral arterial tortuosity is not uncommon, its presence in the brachial artery is relatively rare [17]. To understand the mechanisms that cause tortuosity in the brachial artery, a parallel can be drawn from the study by Schep et al. [20] who conducted a study investigating claudication in endurance cyclists; they found the repetitive flexion and extension of the hip increase tortuosity of the iliac arteries. Notably, symptoms were significantly associated with kinking angles exceeding 70 degrees. In our case, we hypothesize that the lateral root of the median nerve may have acted as a stabilizing anchor via fixing the distal part of the axillary artery by holding its branching common trunk in place. This anatomical tethering likely limited the mobility of the distal axillary artery and imposed a mechanical stress on the proximal brachial artery during arm movements. As the arm elevates, the resulting tension could lead to stretching of the proximal brachial artery, and as the arm returns to a neutral position, the stretched segment may form curvatures. This dynamic relationship may explain the unusual proximal brachial artery morphology.

## Conclusions

This case highlights a novel site of potential dynamic compression of the lateral root of the median nerve by the variant common arterial trunk branch of the axillary artery. Recognition of such anatomical variations and vascular-neural relationships is essential for clinicians assessing upper limb neuropathies and for surgeons operating in the axillary region, as these factors may influence diagnosis, treatment, and surgical outcomes.
